# Potency of integrating three-dimensional cardiac magnetic resonance imaging into electroanatomic mapping to perform catheter ablation in pediatrics

**DOI:** 10.1186/1532-429X-15-S1-M9

**Published:** 2013-01-30

**Authors:** Satoshi Kunimoto, Kensuke Yamamoto, Rie Ichikawa, Junji Fukuhara, Masaharu Matsumura, Naokata Sumitomo, Yasuo Okumura, Ichiro Watanabe, Atsushi Hirayama

**Affiliations:** 1 Division of Cardiology, Nihon University School of Medicine, Tokyo, Japan; 2 Division of Pediatrics, Nihon University School of Medicine, Tokyo, Japan

## Background

Radio-frequency catheter ablation (RFCA) in children is difficult because the size of heart chamber change with age and the catheter made for adults is not appropriate for a child. Three-dimensional (3D) images for electroanatomic mapping (EAM) have been used to create from images of multi-detector computed tomography (MDCT) which need to perform entailed by contrast medium injection and radiation exposure. This study sought to evaluate the usability of 3D Cardiac Magnetic Resonance Image (CMR) in pediatrics cases.

## Methods

Between June 2009 and May 2012, 164 young patients (10.8 ± 5.6 y/o, 89 males) underwent RFCA. 84 patients underwent CMR prior to RFCA without beta blocker and nitroglycerin. CMR was performed by using 1.5-T magnet MR systems (Intera Achieva, Philips Medical Systems) with 5-element cardiac coils. EAM was created by the Carto XP system. CMR results were visually inspected by at least two experienced angiographers whether the image quality of 3D CMR is sufficient for EAM. We also make a comparison of radiation exposure time between with and without 3D CMR images.

## Results

In spite of dull images because of sinus tachycardia, all CMR images were sufficient for EAM. Radiation exposure time with EAM from CMR was significant shorter than without CMR (30.0 ± 2.0 minutes versus 40.6 ± 4.1 minutes, p<0.05).

## Conclusions

CMR image integration into EAM system was successfully performed in pediatrics patients undergoing RFCA. Procedure of CMR didn't require any medication, contrast medium or radiation exposure. In pediatrics cases, almost all cases are considered an indication for CMR imaging for EAM.

## Funding

None.

